# Real-World Evidence of Dydrogesterone 20 mg and 30 mg SR Usage in Pregnancy

**DOI:** 10.7759/cureus.72016

**Published:** 2024-10-21

**Authors:** Madhubala Manickavasagam, Ashish Vakil, Ekika Singh, Himanshu Roy, Nitin Lal, R.G. Patel, Rashi Mishra, Sandeep Gudibanda, Snehal Shah

**Affiliations:** 1 Obstetrics and Gynaecology, Lakshmi Madhavan Hospital, Tirunelveli, IND; 2 Obstetrics and Gynaecology, Vatsalya Hospital and Asha IVF Centre, Una, IND; 3 Obstetrics and Gynaecology, Sharda Narayan Health Care Pvt. Ltd., Mau, IND; 4 Obstetrics and Gynaecology, Srijan Fertility Clinic Pvt. Ltd., Patna, IND; 5 Obstetrics and Gynaecology, Manan Institute For Fertility and Test Tube Baby Centre Pvt. Ltd., Rajkot, IND; 6 Obstetrics and Gynaecology, Sunflower Hospital, Infertility and IVF Center, Ahmedabad, IND; 7 Obstetrics and Gynaecology, Shivani Hospital and IVF Center, Kanpur, IND; 8 CEO, HealthPlix Technologies, Bengaluru, IND; 9 Manager Insights, HealthPlix Technologies, Bengaluru, IND

**Keywords:** dydrogesterone 20 mg sr, dydrogesterone 30 mg sr, electronic medical records (emr), infertility, pregnancy, real-world evidence

## Abstract

Background

Dydrogesterone, an oral selective progesterone receptor agonist with high bioavailability, has been used since the 1960s to treat various conditions arising due to the deficiency of progesterone. Patients are expected to have additional compliance and reduced pill burden with dydrogesterone 20 mg and 30 mg SR once a day (OD) for conditions requiring 10 mg twice a day (BID) or thrice a day (TID).

Methodology

A real-world analysis was conducted using the HealthPlix electronic medical records (EMR) database to understand the demography, indications, and prescription patterns of dydrogesterone 20 mg and 30 mg SR, including co-prescriptions.

Results

The mean age of the female patients prescribed dydrogesterone 20 mg SR was 27.23 ± 4.79 years while those prescribed 30 mg SR had a mean age of 28.56 ± 5.17 years. Common indications for the prescription of dydrogesterone 20 mg SR were pregnancy, infertility, and abortion/miscarriage. Pregnancy, infertility, and amenorrhea were the common conditions for dydrogesterone 30 mg SR prescription. The average duration of prescription of dydrogesterone 20 mg and 30 mg SR was 55.50 ± 31.33 and 79.66 ± 68.38 days, respectively. OD regimen was the preferred regimen for dydrogesterone 20 mg SR (89.48%) and dydrogesterone 30 mg SR (64.06%).

Conclusions

This analysis suggests dydrogesterone 20 mg and 30 mg SR are being prescribed for various indications in the real world with significant variation in the prescription patterns.

## Introduction

Progesterone, an endogenous steroid, has a key physiological role in the menstrual cycle, reproduction, and steroid hormone biosynthesis [1]. It is responsible for creating an endometrial environment suitable for the implantation of an embryo and subsequently maintenance of pregnancy [2,3]. During the luteal phase and early phase of the first trimester, this is produced by the corpus luteum. The placenta takes over this function between 7 and 11 weeks of pregnancy [4].

Inadequate production of progesterone by the corpus luteum after ovulation is associated with infertility. In the case of conception, progesterone levels rise rapidly in the early luteal phase and attain higher mid-luteal levels compared with menstrual cycles without successful conception. Insufficient levels of progesterone post-implantation can result in miscarriage, the most common complication of early pregnancy [5,6]. Progesterone supplementation in such cases can help reduce the risk of miscarriage in females. The use of steroid hormones during the first trimester can be fatal because organogenesis occurs during this phase and there is a plausibility of congenital anomalies. Therefore, synthetic progestogens (progestins) such as dydrogesterone with different molecular structures, pharmacodynamics, pharmacokinetics, and safety profiles are often prescribed [7]. These mimic the role of progesterone by binding to the progesterone receptor and are prescribed for different gynecological and obstetric conditions [8,9].

Dydrogesterone, an oral retrosteroid closely related to progesterone, has a higher bioavailability and selectivity for progesterone receptors. Its bent shape and retro structure result in 5.6 times higher bioavailability than progesterone [4]. Ingale et al. conducted a survey to understand the prescription pattern of micronized progesterone and dydrogesterone in pregnancy and assisted reproductive technology cycles. It was reported that 35.8% of gynecologists prescribed dydrogesterone 10 mg thrice a day (TID) to prevent preterm labor in case of twin pregnancy [10]. In a knowledge, attitude, and practice survey by Khanna et al., dydrogesterone 10 mg twice daily (BID) was the most recommended schedule (73% of gynecologists) [11]. The Lotus I trial was conducted to compare the efficacy, safety, and tolerability of oral dydrogesterone 30 mg (10 mg TID) against micronized vaginal progesterone 600 mg daily (200 mg TID) for luteal support in in-vitro fertilization (IVF). The study demonstrated a favorable benefit/risk profile and adequate evidence that dydrogesterone is at par with the present standard of care for females undergoing IVF [12]. A study was conducted by Sasikala et al. to compare the efficacy and safety of dydrogesterone 20 mg extended-release (ER) once daily (OD) versus dydrogesterone 10 mg BID in patients diagnosed with endometriosis. It was reported that dydrogesterone 20 mg ER and dydrogesterone 10 mg exhibited a similar and significant reduction in endometriosis-associated pelvic pain score and other parameters. Considerable improvements were reported in the parameters associated with quality of life [13]. Dosage frequency is known to have a strong impact on patient compliance [14]. Multiple daily dosing may be negatively associated with patient compliance and adherence to medications [15]. Compliance is expected to improve in the case of the lowest daily recommended dose frequency [16].

Considering this an important factor for adherence and compliance, pharmaceutical companies conceptualized developing dydrogesterone 20 mg and 30 mg sustained release (SR) for the conditions where physicians would prescribe 10 mg BID or TID, respectively. There is limited real-world data on the usage of dydrogesterone 20 mg and 30 mg SR. Hence, this study was conducted to understand the prescription pattern of dydrogesterone 20 mg and 30 mg SR.

## Materials and methods

This was a real-world, retrospective, electronic medical records (EMR)-based study. Anonymized and aggregated data of patients meeting the eligibility criteria for the period January 2023 to June 2024 were retrieved from the EMR database of HealthPlix Technologies Private Limited (https://www.healthplix.com/). HealthPlix is a platform currently used by more than 14,000 doctors for writing prescriptions. Female patients aged ≥18 years who were prescribed dydrogesterone 20 mg or 30 mg SR were included in the study. Patients who were prescribed dydrogesterone 10 mg were excluded. The data were retrieved in an Excel format and cleaned as per the HealthPlix standard operating procedure. Patient confidentiality was maintained throughout the study, and the data extracted for the study did not have any patient identifiers. The study was approved by the Royal Pune Ethics Committee on August 28, 2024 (approval number: RPIEC140824).

The primary endpoint of the study was to evaluate the indications for the prescription of dydrogesterone 20 mg and 30 mg SR. Secondary endpoints were to assess the average prescription duration of dydrogesterone 20 mg and 30 mg SR, frequency of daily prescription, and the co-prescribed medications. The average prescription duration of dydrogesterone 20 mg and 30 mg SR for pregnancy was also assessed as an exploratory endpoint. Analysis of indications and co-prescribed medications was done using the data from the visit when dydrogesterone 20 mg or 30 mg SR was prescribed. For evaluation of the average prescription duration, the total duration of prescription across all visits on the EMR platform was considered (this was evaluated for the ones who had at least one follow-up visit after the prescription of dydrogesterone 20 mg or 30 mg SR).

Stata version 15.1 SE (StataCorp., College Station, TX, USA) was used for performing the statistical analysis for this study. Indications were presented as the number and percentage of patients. Similarly, the co-prescribed medications were presented as numbers and percentages. The average duration of prescription (days) was represented as the mean ± standard deviation (SD). This was also separately evaluated for pregnant females, presented as mean ± SD. The daily recommendation of dydrogesterone prescription was summarized as the number and percentage of patients.

## Results

Patient disposition

**Figure 1:**
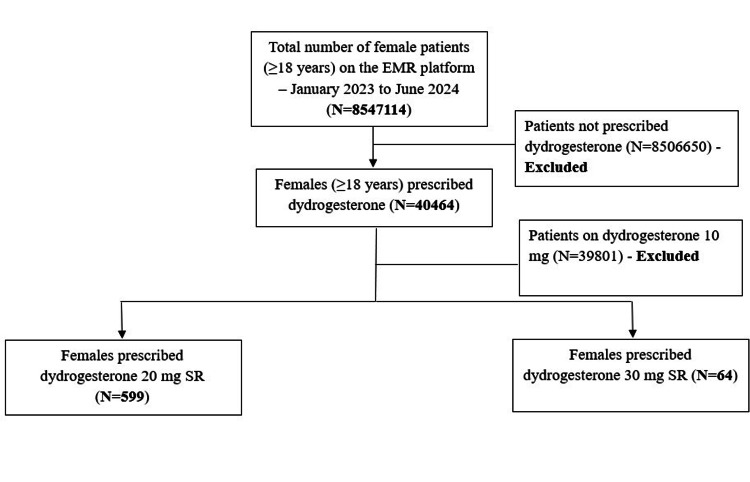
Flowchart showing patient disposition.

There were records of 85,47,114 female patients (≥18 years) on the EMR platform for the period of January 2023 to June 2024, of whom 40,464 were prescribed dydrogesterone. Dydrogesterone 20 mg SR and 30 mg SR were prescribed to 599 and 64 patients respectively (Figure 1).

Demographic characteristics

Dydrogesterone 20 mg SR was prescribed to 599 patients, and the mean age for these patients was 27.23 ± 4.79 years. The mean age of the patients prescribed dydrogesterone 30 mg SR (N = 64) was 28.56 ± 5.17 years. Table 1 presents the age characteristics of these patients.

**Table 1:** Demographic characteristics (age). SR: sustained release; SD: standard deviation; Min: minimum; Max: maximum

Dose strength	Patient count	Mean ± SD (years)	Min, Max (years)
Dydrogesterone 20 mg SR	599	27.23 ± 4.79	18, 46
Dydrogesterone 30 mg SR	64	28.56 ± 5.17	18,41

Indications for prescription

Dydrogesterone 20 mg SR

On analysis of the real-world data for dydrogesterone 20 mg SR, the highest proportion of prescriptions was for pregnancy (55.26%). Of the pregnant females (n = 331), further categorization of pregnancy was not specified for 71.90% of the females, 22.05% were in the early phase of pregnancy, and 3.32% were in the very early phase. This dose strength of dydrogesterone was also prescribed in cases of ectopic pregnancy, twin pregnancy, and precious pregnancy. Dydrogesterone 20 mg SR was prescribed to 40 patients with a diagnosis of infertility. Of these, 52.50% were cases of primary infertility and 37.50% of secondary infertility. Patients with abortion/miscarriage (n = 12) were also prescribed this medication. Most of these cases (50%) were threatened abortion. Other indications observed were polycystic ovarian disease (PCOD), adenomyosis, cysts, and others (Table 2).

**Table 2:** Indications for prescription of dydrogesterone 20 mg SR. The percentage of patients for indications was calculated by taking 599 (total number of patients prescribed dydrogesterone 20 mg SR) as the denominator. Percentage for sub-classification of indications was calculated based on the number of patients with the indication. Sub-classification of indications has been provided based on the diagnosis mentioned by doctors in the prescriptions. PCOD: polycystic ovarian disease; PCOS: polycystic ovarian syndrome; IVF: in vitro fertilization

	Indication	Number of patients (n = 599)	Percentage (%)
Pregnancy-related conditions	Pregnancy	331	55.26
	Pregnancy	238	71.90
	Early pregnancy	73	22.05
	Very early pregnancy	11	3.32
	Ectopic pregnancy	2	0.60
	Twin pregnancy	2	0.60
	Precious pregnancy	1	0.30
	Triplets pregnancy	1	0.30
	Delayed pregnancy	1	0.30
	Chemical pregnancy	1	0.30
	Biochemical pregnancy	1	0.30
	Infertility	40	6.68
	Primary infertility	21	52.50
	Secondary infertility	15	37.50
	Infertility	2	5.00
	Secondary subfertility	2	5.00
	Abortion/Miscarriage	12	2.00
	Threatened abortion	6	50.00
	Missed abortion	3	25.00
	Recurrent abortion	1	8.33
	Spontaneous abortion	1	8.33
	Threatened miscarriage	1	8.33
	PCOD	8	1.34
	PCOS	1	0.17
	IVF	1	0.17
Others	Adenomyosis	5	0.83
	Cyst	5	0.83
	Endometriosis	1	0.17
	Fibroid	1	0.17
	Missing diagnosis/Miscellaneous	194	32.39

Dydrogesterone 30 mg SR

Pregnancy was the most common indication (46.88%) for the prescription of dydrogesterone 30 mg SR. The stage of pregnancy was not defined for 73.33% of pregnant females (n = 30). Infertility was an indication for eight (12.50%) of those advised dydrogesterone 30 mg SR. This dose strength was also recommended for indications such as amenorrhea, IVF, PCOD, endometriosis, fibroid, and others mentioned (Table 3).

**Table 3:** Indications for prescription of dydrogesterone 30 mg SR. The percentage of patients for indications was calculated by taking 64 (total number of patients prescribed dydrogesterone 30 mg SR) as the denominator. Percentage for sub-classification of indications was calculated based on the number of patients with the indication. Sub-categories of indications have been provided based on the diagnosis mentioned by doctors in the prescriptions. IVF: in vitro fertilization; PCOD: polycystic ovarian disease

	Indication	Number of patients (n = 64)	Percentage (%)
Pregnancy-related conditions	Pregnancy	30	46.88
	Pregnancy	22	73.33
	Early pregnancy	7	23.33
	Elderly pregnancy	1	3.33
	Infertility	8	12.50
	Infertility	1	12.50
	Primary infertility	6	75.00
	Secondary infertility	1	12.50
	Amenorrhea	5	7.81
	IVF	5	7.81
	PCOD	3	4.69
	Ruptured follicles	3	4.69
	Abortion	2	3.13
	Missed abortion	2	100
Others	Endometriosis	1	1.56
	Fibroid	1	1.56
	Missing diagnosis/Miscellaneous	6	9.38

Average duration of prescription

The average duration of prescription was calculated for patients who had at least one follow-up visit after the prescription of dydrogesterone 20 mg or 30 mg SR. In the case of patients prescribed dydrogesterone 20 mg SR (n = 230), the mean duration of prescription was 55.50 ± 31.33 days, and in the case of dydrogesterone 30 mg SR (n = 27), this was 79.66 ± 68.38 days. Table 4 summarizes the average duration of prescription of dydrogesterone 20 mg and 30 mg SR.

**Table 4:** Average duration of prescription of dydrogesterone 20 mg and 30 mg SR. Average duration of prescription was calculated for the ones who had at least one follow-up visit after the prescription of dydrogesterone 20 mg or 30 mg SR. SR: sustained release; SD: standard deviation; Min: minimum; Max: maximum

Dose strength	Patient count	Mean ± SD (days)
Dydrogesterone 20 mg SR	230	55.50 ± 31.33
Dydrogesterone 30 mg SR	27	79.66 ± 68.38

Average duration of prescription of dydrogesterone 20 mg and 30 mg SR for pregnancy

The average duration of prescription of dydrogesterone 20 mg and 30 mg SR in case of pregnancy was 57.21 ± 28.78 and 63.86 ± 62.99 days, respectively (Table 5).

**Table 5:** Average duration of prescription of dydrogesterone 20 mg and 30 mg SR for pregnancy. Average duration of prescription for pregnancy was calculated for pregnant females who had at least one follow-up visit after the prescription of dydrogesterone 20 mg or 30 mg SR. SR: sustained release; SD: standard deviation; Min: minimum; Max: maximum

Dose strength	Patient count	Mean ± SD (days)
Dydrogesterone 20 mg SR	139	57.21 ± 28.78
Dydrogesterone 30 mg SR	30	63.86 ± 62.99

Daily recommendation

Table 6 presents the daily recommended frequency for dydrogesterone 20 mg and 30 mg SR. The most common regimen of prescription was OD. This schedule was observed for 89.48% of the patients on dydrogesterone 20 mg SR and 64.06% on dydrogesterone 30 mg SR.

**Table 6:** Daily recommendation of dydrogesterone 20 mg and 30 mg SR. N: total number of patients prescribed dydrogesterone 20 mg or 30 mg SR; n: number of patients prescribed medications for different frequencies; OD: once a day; BID: twice a day; TID: thrice a day; NA: not available

Dose strength	N	OD, n (%)	BID, n (%)	TID, n (%)	NA, n (%)
Dydrogesterone 20 mg SR	599	536 (89.48)	51 (8.51)	6 (1.00)	6 (1.00)
Dydrogesterone 30 mg SR	64	41 (64.06)	22 (34.38)	-	1 (1.56)

Co-prescribed medications

Dydrogesterone 20 mg SR

Amongst the co-prescribed hormonal preparations, progesterone was most prescribed (17.20%), followed by human chorionic gonadotropin (12.02%). The combination of l-methylfolate + mecobalamin + vitamin B6 was the most frequently prescribed vitamin supplement (8.35%). Elemental iron was prescribed to 2.34% of the patients recommended dydrogesterone 20 mg. Medications co-prescribed with dydrogesterone 20 mg SR are presented in Table 7.

**Table 7:** Medications co-prescribed with dydrogesterone 20 mg SR. The percentage of patients for each medication was calculated by taking the total number of patients prescribed dydrogesterone 20 mg SR as the denominator. SR: sustained release

Therapy and medication	Number of patients (n)	Percentage (%)
Hormonal preparations		
Progesterone	103	17.20
Human chorionic gonadotropin	72	12.02
Hydroxyprogesterone caproate	30	5.01
Chorionic gonadotropin	5	0.83
Estradiol	1	0.17
Medroxyprogesterone acetate	1	0.17
Vitamin supplements		
l-methylfolate + mecobalamin + vitamin B6	50	8.35
Biotin + cholecalciferol + ferrous ascorbate + l-arginine + l-leucine + l-methylfolate calcium + manganese sulphate + methylcobalamin + potassium iodide + vitamin B6 + zinc sulphate	6	1.00
Cholecalciferol + chromium picolinate + d-chiro-inositol + myo-inositol	1	0.17
Iron and its supplements		
Elemental iron	14	2.34
Ferrous ascorbate + folic acid	10	1.67
Ferrous fumarate + folic acid + zinc sulfate	4	0.67
Ferrous ascorbate + l-methylfolate	4	0.67
Ferrous chelate + folic acid + vitamin B12 + zinc picolinate	2	0.33
Smooth muscle relaxants		
Isoxsuprine	4	0.67
Drugs for high prolactin levels		
Cabergoline	1	0.17

Dydrogesterone 30 mg SR

In these patients too, progesterone was the most prescribed (50%). Estradiol and human chorionic gonadotropin were prescribed to 12.50% and 3.13% of the patients, respectively. Table 8 presents the medications co-prescribed with dydrogesterone 30 mg SR.

**Table 8:** Medications co-prescribed with dydrogesterone 30 mg SR. The percentage of patients for each medication was calculated by taking the total number of patients prescribed dydrogesterone 30 mg SR as the denominator. SR: sustained release

Therapy and medication	Number of patients (n)	Percentage (%)
Hormonal preparations		
Progesterone	32	50.00
Estradiol	8	12.50
Human chorionic gonadotropin	2	3.13
Vitamin supplements		
Lactic acid + sea buckthorn oil + tea tree extract	1	1.56
Astaxanthin + elemental zinc + folic acid + l-arginine + lycopene + vitamin B12 + vitamin B6	1	1.56
Iron and its supplements		
Ferrous fumarate + folic acid + vitamin B12	1	1.56
Calcium + choline + docosahexaenoic acid + iron + potassium + vitamin B1 + vitamin B2 + vitamin B6 + vitamin C + zinc	1	1.56
Elemental iron + l-methylfolate	1	1.56

## Discussion

Dydrogesterone, an orally active synthetic progestogen with a structure similar to that of natural progesterone, can be used by females throughout their lifetime, i.e., from adolescence to older age [17]. In our study, the mean age of females prescribed dydrogesterone 20 mg SR and dydrogesterone 30 mg SR was 27.23 ± 4.79 and 28.56 ± 5.17 years, respectively. Tank et al. assessed the real-world utilization pattern of dydrogesterone in Indian women with obstetric and gynecological conditions. The mean age of the included population was 29.55 ± 4.84 years [18]. The mean age of females in our study was similar to the one observed in the published literature.

Dydrogesterone is a crucial component of menopausal hormone therapy. In younger females, this is primarily indicated for the treatment of dysmenorrhea, irregular menstrual cycles, premenstrual syndrome, and threatened or recurrent miscarriage. It is also known to be effective as luteal phase support during assisted reproduction techniques [17]. Pregnancy was the most common indication in the case of both dydrogesterone 20 mg and 30 mg SR. It was also prescribed for infertility, abortion, PCOD, polycystic ovarian syndrome (PCOS), IVF, adenomyosis, endometriosis, and others. Miscarriage, spontaneous loss of pregnancy before 24 weeks of gestation, is a common complication of pregnancy and 15% to 20% of pregnancies end up in miscarriage. Progesterone plays a crucial role in the implantation and maintenance of pregnancy. Progesterone supplementation is generally advised for females with bleeding episodes during early pregnancy (threatened abortion). It is also recommended for asymptomatic women who have a history of three or more previous miscarriages to prevent loss of pregnancy [19]. Inadequate production of progesterone by the corpus luteum post-ovulation is generally associated with infertility and is often treated with progestogens which emulate the activity of progesterone by extending luteal phase support [4]. The literature suggests that dydrogesterone is prescribed to patients with endometriosis. It causes atrophy of the ectopic endometrial tissue without suppressing the normal endometrium and inhibits the development of new endometriotic lesions [20,21]. Patients were recommended dydrogesterone 20 mg or 30 mg SR for a duration deemed appropriate by their physicians for that condition. The average duration of prescription for dydrogesterone 20 mg SR was 55.50 ± 31.33 days, whereas for dydrogesterone 30 mg SR, it was 79.66 ± 68.38 days. In the case of pregnancy, this duration was 57.21 ± 28.78 and 63.86 ± 62.99 days for dydrogesterone 20 mg SR and dydrogesterone 30 mg SR, respectively. In case of threatened abortion, the recommendations mention that a loading dose of up to 40 mg may be given followed by 20 mg or 30 mg per day till the symptoms remit. Dydrogesterone is prescribed 10 mg BID until the 20th week of pregnancy for recurrent pregnancy loss. For the treatment of infertility due to luteal insufficiency, dydrogesterone is prescribed 10 or 20 mg daily starting with the second half of the menstrual cycle until the first day of the next cycle for three consecutive cycles. In cases of endometriosis, dydrogesterone is recommended at a dose of 10 mg to 30 mg daily from day 5 to 25 of the cycle or continuously. Further, 10 or 20 mg daily beginning with the second half of the menstrual cycle until the first day of the next cycle is recommended for irregular cycles. For premenstrual syndrome, females are prescribed 10 mg daily starting with the second half of the menstrual cycle until the first day of the next cycle [18]. OD regimen was observed to be the most common for the patients included in this study. Sasikala et al. conducted a safety and efficacy study of OD dydrogesterone 20 mg ER versus dydrogesterone 10 mg BID in Indian patients with endometriosis. They reported that dydrogesterone 20 mg ER had similar efficacy and safety compared to BID dydrogesterone 10 mg [13]. It is known that compliance is inversely related to the number of doses per day [22]. This indicates that patients are expected to have better compliance in case of treatment with dydrogesterone 20 mg or 30 mg SR which are generally prescribed with simpler and less frequent dosing regimens. The most common medication co-prescribed with dydrogesterone 20 mg and 30 mg SR was progesterone. This was prescribed to 17.20% and 50.00% of the patients on dydrogesterone 20 mg SR and 30 mg SR, respectively. Human chorionic gonadotropin was prescribed to 12.02% of patients on dydrogesterone 20 mg SR and 3.13% of patients on dydrogesterone 30 mg SR. Elemental iron, a combination of ferrous ascorbate and folic acid, was advised to 2.34% and 1.67% of the patients on 30 mg SR and 20 mg SR dose strengths, respectively. Tank et al. reported folic acid, iron supplements, and calcium and vitamin D3 supplements as the most commonly co-prescribed. Progesterone preparations were used by 7.8% of the subjects [18].

## Conclusions

Progesterone deficiency is generally associated with infertility and miscarriage. Progestogens which mimic the role of progesterone are prescribed in such cases. Physicians usually prescribe dydrogesterone 10 mg BID/TID. Daily frequency is known to have a significant effect on patient compliance: the less the frequency, the better the compliance. Considering this, the new dose strengths, i.e., 20 mg SR and 30 mg SR were launched, and this study was conceptualized. EMR-based data were retrieved to understand the indications, average prescription duration, and daily recommendations. Analysis of the real-world data suggests that dydrogesterone 20 mg and 30 mg SR are prescribed for a broad spectrum of indications. OD was the most preferred recommendation with an average duration of 57.21 ± 28.78 days for dydrogesterone 20 mg SR and 79.66 ± 68.38 days for dydrogesterone 30 mg SR. The current prescribing practice suggests that these dose strengths would be helpful in the case of patients requiring 10 mg BID or TID.
